# Antioxidants and breast cancer risk- a population-based case-control study in Canada

**DOI:** 10.1186/1471-2407-11-372

**Published:** 2011-08-24

**Authors:** Sai Yi Pan, Jia Zhou, Laurie Gibbons, Howard Morrison, Shi Wu Wen

**Affiliations:** 1 Centre for Chronic Disease Prevention and Control, Public Health Agency of Canada, Ottawa, Ontario, Canada; 2 Ottawa Institute of Health Research, Ottawa, Ontario, Canada

**Keywords:** breast cancer, case-control study, antioxidants, supplementation, dietary intake

## Abstract

**Background:**

The effect of antioxidants on breast cancer is still controversial. Our objective was to assess the association between antioxidants and breast cancer risk in a large population-based case-control study.

**Methods:**

The study population included 2,362 cases with pathologically confirmed incident breast cancer (866 premenopausal and 1,496 postmenopausal) and 2,462 controls in Canada. Intakes of antioxidants from diet and from supplementation as well as other potential risk factors for breast cancer were collected by a self-reported questionnaire.

**Results:**

Compared with subjects with no supplementation, 10 years or longer supplementation of zinc had multivariable-adjusted odds ratios (OR) and 95% confidence intervals (CI) of 0.46 (0.25-0.85) for premenopausal women, while supplementation of 10 years or longer of multiple vitamin, beta-carotene, vitamin C, vitamin E and zinc had multivariable-adjusted ORs (95% CIs) of 0.74 (0.59, 0.92), 0.58 (0.36, 0.95), 0.79 (0.63-0.99), 0.75 (0.58, 0.97), and 0.47 (0.28-0.78), respectively, for postmenopausal women. No significant effect of antioxidants from dietary sources (including beta-carotene, alpha-carotene, lycopene, lutein and zeaxanthin, vitamin C, vitamin E, selenium and zinc) or from supplementation less than 10 years was observed.

**Conclusions:**

This study suggests that supplementation of zinc in premenopausal women, and supplementation of multiple vitamin, beta-carotene, vitamin C, vitamin E and zinc in postmenopausal women for 10 or more years may protect women from developing breast cancer. However, we were unable to determine the overall effect of total dose or intake from both diet and supplement.

## Background

Breast cancer is the most common cancer and the second leading cause of cancer-related death among Canadian women [1]. According to the most recent projection from Canadian Cancer Society, there will be 22,700 new cases of female breast cancer representing 27.9% of all cancer cases in women, and 5,400 deaths caused by breast cancer accounting for 15.1% of all cancer-related deaths in women in Canada in 2009 [1].

Recently, antioxidants such as vitamin A and its precursor (beta-carotene, lycopene, lutein, etc), vitamins C, E, and selenium, have drawn a lot of attention to the scientists and the public alike [2,3]. They have been shown in experimental studies to neutralize or trap reactive oxygen species (also known as free radicals), thereby preventing cellular damage caused by the reaction of these species with proteins and nucleic acids [4-9]. Inflammation, infection, extreme exercise and exposure to various environmental factors, including pollution, tobacco smoke and radiation, can increase the level of free radicals in the body [3]. Excess production of free radicals and/or deficiency of the antioxidant defence system can result in oxidative stress, causing damage to DNA and other molecules [4,5]. Over time, such damage may become irreversible and may lead to diseases such as cancer. There is evidence showing that oxidative stress and lipid peroxidation are linked to the aetiology of breast cancer [5]. Antioxidants are often described as "mopping up" free radicals, therefore they decrease oxidative stress and oxidative DNA damage [4,6,7,9]. It has also been shown that antioxidants may selectively induce apoptosis in cancer cells but spare normal cells, and inhibit cell proliferation [3,10,11].

Although there is considerable evidence of anticancer effect of antioxidants from cell culture and animal studies, the results from observational studies and intervention trials are inconsistent [8,12,13]. A recent review conducted by Michels et al summarized the findings from prospective observational studies of the role of diet on the risk of breast cancer, including antioxidants, and found that the reported results were not consistent and sometimes controversial regarding the association between breast cancer incidence and any dietary antioxidants and blood antioxidants [2]. Randomized clinical trials of the effects of antioxidants on chronic diseases including breast cancer, also reached inconsistent conclusions [8,13,14]. However, since antioxidant enhancement, either through increased dietary intake or by supplementation, is a preventive measure that can be easily implemented, further studies to look at this association are warranted. The objective of this study was to assess the association between antioxidants from both diet and supplement and breast cancer, using data from a large population based case-control study in Canada.

## Methods

### Study population

This study was based on data collected by the National Enhanced Cancer Surveillance System (NECSS). The details of NECSS, including the study design, study population, and data collection strategies, have been published elsewhere [15], and are briefly described here. The NECSS was a multi-component, collaborative project of Health Canada and the provincial cancer registries. The case-control component included individual data from 21,020 Canadians with one of 19 types of cancers (including 2362 cases with breast cancer) and 5,039 population controls (including 2492 females and 2647 males) aged 20- 76 years, which were collected between 1994 and 1997 in eight of the 10 Canadian provinces (Alberta, British Columbia, Manitoba, Newfoundland, Nova Scotia, Ontario, Prince Edward Island and Saskatchewan). The respective ethics review boards of each province reviewed and approved the study proposal. The current analysis was based on 2,362 incident cases of breast cancer (866 premenopausal and 1,496 postmenopausal) and 2,462 controls from all eight provinces.

The population-based provincial cancer registries identified breast cancer cases through a review of pathology reports. All cases were pathologically confirmed incident breast cancer patients, newly diagnosed between 1994 and 1997 in the eight participating provinces. Breast cancer was defined as C50 according to the *International Classification of Diseases for Oncology*, Second Edition (ICD-O-2). The cancer registries tried to identify cases as soon as possible after diagnosis to reduce the loss of subjects caused by severe illness and death. Provincial cancer registries identified 3386 women with breast cancer. Physician consent to contact the cases was requested for 3248 cases. Seventy-four women had died at the time of the physician request, and the physician refused consent for another 224 cases. Questionnaires were sent to 3013 cases and 2982 cases were contacted. Completed questionnaires were received from 2362 cases, representing 78.4% of cases who were sent questionnaires and 69.7% of those ascertained.

Control subjects were women with no prior diagnosis of cancer and residents of the participating provinces. In NECSS, frequency matching to the overall case group (19 types of cancers) was used to select population controls with similar age and sex distribution, so that there would be at least one control for every case within each sex and 5-year age group for any specific cancer site within each province. The sampling strategy for control selection varied by province, depending on data availability, data quality (completeness and timeliness) and the confidentiality restrictions of provincial databases. Prince Edward Island, Nova Scotia, Manitoba, Saskatchewan, and British Columbia used provincial health insurance plans to get a random sample of the provincial population stratified by age group and sex. More than 95% Canadians are covered by these public plans, and individuals are excluded only if covered through other federal plans. In Ontario, the Provincial Ministry of Finance Property Assessment databases, which are intended to include all residents of the province and are updated monthly, were used to obtain a stratified random sample. Newfoundland and Alberta used similar random digit dialling protocols to obtain population samples.

Questionnaires were mailed to 3847 women selected as potential controls. For 286 of these women 7.4%, the mailed questionnaires were returned to the registry indicating a wrong or old address, and no updated address could be found through publicly available sources. In total, 2492 women completed and returned the questionnaire, representing 71.3% of those contacted and 64.8% of these ascertained.

### Data collection

The provincial registries collected data by self-administered questionnaires, with telephone follow-up when necessary for clarification and completeness. The questionnaires were designed to obtain detailed data on potential risk factors for cancers. The questionnaires collected information on education, average family income over the last 5 years, marital status, ethnic group, smoking, height, weight, physical activity, alcohol consumption, diet (69-item food frequency questions) 2 years before interview, and length of vitamin and mineral supplements for the past 20 years. Questionnaires also gathered information about menstrual and reproductive history and residential history.

### Assessment of dietary intake

The diet component of the questionnaire used a 69-item food frequency instrument to collect information on diet from 2 years before the interview date. The design of the diet component of the questionnaire was based on two validated instruments: the National Cancer Institute's Block Questionnaire [16], and the instrument used in the Nurses' Health Study cohort [17], with minor modifications to account for the differences between the Canadian and American diet. These two instruments have been widely used in studies on diet and cancer.

A commonly used portion or serving size was specified for each food or beverage item. Respondents indicated the usual frequency of consumption of that portion size for each food item by selecting one of nine possible categories: 0 or < 1 per month, 1 to 3 per month, 1 per week, 2 to 4 per week, 5 to 6 per week, 1 per day, 2 to 3 per day, 4 to 5 per day, or ≥ 6 per day. The weekly consumption of each food item was then calculated as the product of frequency and serving size. The nutrient content of foods was determined using food composition data from the Canadian Nutrient Guide and Canadian Nutrient File [18]). The total weekly intake level of each nutrient was determined by multiplying the weekly consumption quantity of each food item by its associated nutrient value, and calculating the sum of the weekly nutrient intake levels for all 69 food items.

The questionnaire also collected information regarding vitamin or mineral supplement use (frequency and length) during the past 20 years. Respondents were asked how often (never, not regularly, and fairly regularly) and for how many years in total they had taken each of the 10 vitamins and mineral supplements (less than 1 year, 1-2 years, 3-5 years, 6-9 years, 10-19 years, and 20+ years). The ten vitamins and minerals are multiple vitamins, vitamin A, vitamin C, vitamin E, B-complex vitamins, beta-carotene, calcium, iron, zinc, and selenium.

### Assessment of other variables

The questionnaire gathered information on recreational physical activity 2 years before interview. Respondents were asked in which seasons, how often and how long per session, on average, they participated in each of the 12 most common types of leisure-time physical activity in Canada. Individual activities included walking for exercise, bowling or curling, social dancing, gardening or yard work, golf, home exercise or exercise class, bicycling, jogging or running, racquet sports, swimming or water exercise, skiing or skating, and other strenuous exercise. Respondents indicated their usual frequency of participating in each of the above activities by choosing one of the following categories: never, less than once per month, 1 - 3 times per month, 1 - 2 times per week, 3 - 6 times per week or every day.

As a measure of overweight and obesity, body mass index (BMI) was calculated as the reference weight in kilograms (2 years before interview) divided by height in meters squared.

### Statistical analysis

We estimated the risk of breast cancer associated with intake of dietary antioxidants (beta-carotene, lycopene, lutein, vitamin C, vitamin E, and selenium) and with years of supplementation of antioxidants (multiple vitamin, vitamin A, beta-carotene, vitamin C, vitamin E, and selenium) based on odds ratios (ORs) and corresponding 95% confidence intervals (CIs), using unconditional logistic regression with the software package SAS (version 9; SAS Institute, Inc., Cary, North Carolina). For antioxidants from dietary intake, exposure variables were categorized into quartiles based on the distribution of the variables in the control population, with the lowest quartile as the reference. For antioxidants from supplementation, exposure variables were categorized by length of supplementation (never taken, ≤ 2 years, 3-9 years, and ≥ 10 years, with never taken as the reference). Total intake of antioxidants was examined in following way: high (equal to or high than median) versus low (lower than median) intake of antioxidant from diet only, high (≥ 10 years) versus low (< 10 years) intake from supplementation only, and high intake from both diet (equal to or high than median) and supplementation (≥ 10 years) versus low intake (from either sources). The analysis was performed for premenopausal and postmenopausal breast cancers separately. We conducted tests for trends for all models of categorized data by treating the different categories as a single ordinal variable.

Because cases and controls were not directly matched, the methods for identifying cases and controls varied by province; and, because age is associated with breast cancer risk, all logistic regression analyses were controlled for province of residence and age to remove the impact of any uneven distribution of these factors between cases and controls. We used the change-in-point-estimate approach to assess the potential confounding effect of a wide range of factors, including age, family income, educational level, alcohol consumption, smoking, BMI, total calorie intake, recreational physical activity level, menopausal status, and number of live births. We retained variables in the final models that are considered biologically important if their inclusion changed the odds ratio estimate appreciably, regardless of the statistical significance. We adjusted the final multivariate models for age (years, continuous), province of residence, education (years, continuous), number of live births (none, 1, 2, 3, and > = 4), age at menarche (years, continuous), alcohol consumption (servings per week, continuous), pack-years of smoking (continuous) and total calorie intake (kilocalories per week, continuous). For postmenopausal women, the models were also adjusted for BMI and recreational physical activity. Mutual adjustments were done for analyses on antioxidants from dietary source and from supplementation.

## Results

Data from 2,362 breast cancer cases (866 premenopausal cases and 1,496 postmenopausal cases) and 2,462 controls were available for analysis.

Table 1 shows the distribution of some selected characteristics of breast cancer cases and controls. For premenopausal women, compared with controls, cases were slightly older and had less live birth and longer years of menstruation. For postmenopausal women, compared with controls, cases were slightly younger and had higher education, longer smoking pack-years, higher alcohol consumption, higher BMI, less live births, and longer years of menstruation.

**Table 1:** Comparison of cases and controls on demographics and potential confounding factors, NECSS, Canada, 1994-1997

	Premenopausal			Postmenopausal		
		
Characteristic	Cases (N = 866)	Controls (N = 845)	*P*	Cases(1496)	Controls (n = 1617)	*P*
Age (mean ± SD)	44.8 ± 6.2	43.8 ± 7.6	0.003	62.0 ± 8.5	62.7 ± 8.1	0.03
Education (years) (mean ± SD)	13.5 ± 3.1	13.6 ± 3.3	0.51	11.7 ± 3.4	11.3 ± 3.5	0.0009
Smoking pack-years (mean ± SD)	5.9 ± 9.4	5.8 ± 9.4	0.72	10.5 ± 15.0	8.8 ± 14.4	0.001
Alcohol consumption (drinks/wk) (mean ± SD))	2.9 ± 5.7	2.7 ± 5.1	0.48	3.1 ± 6.7	2.5 ± 5.6	0.01
BMI (kg/m^2 ^) (mean ± SD)	24.7 ± 5.2	24.5 ± 4.8	0.29	26.1 ± 5.9	25.7 ± 5.5	0.03
Moderate physical Activity (frequency/month (mean ± SD))	15.9 ± 14.9	15.7 ± 14.1	0.67	18.6 ± 15.4	18.0 ± 15.9	0.28
Strenuous physical activity (frequency/month (mean ± SD))	4.8 ± 8.4	4.9 ± 8.0	0.77	3.3 ± 7.8	3.5 ± 8.1	0.42
Total energy intake (kcals/week)	12915 ± 4791	12687 ± 5440	0.35	12965 ± 5333	12775 ± 6997	0.39
Years of menstruation (mean ± SD)	32.0 ± 6.2	30.9 ± 7.7	0.001	33.0 ± 7.7	32.4 ± 7.6	0.02
Age at menarche (mean ± SD)	12.6 ± 1.4	12.8 ± 1.5	0.005	12.8 ± 1.6	13.0 ± 1.7	0.01
Marital status			0.89			< 0.0001
Marred	659	621		1003	1057	
Common law	51	44		19	27	
Divorced/separated	74	97		136	129	
Windowed	16	18		248	340	
Single	60	59		86	59	
Other	4	4		2	2	
No. live birth			0.27			< 0.0001
0-	162	168		230	174	
1-	118	107		153	143	
2-	366	330		400	395	
3-	162	146		338	319	
≥ 4	56	93		372	581	
Family income adequacy			0.05			0.17
Low	102	111		283	306	
Low middle	146	138		234	276	
Upper middle	274	259		302	317	
High	202	155		217	199	
Smoking status			0.72			0.006
Never	409	413		697	826	
Ex-smoker	267	221		488	505	
Current smoker	190	211		311	286	

Table 2 shows the average intakes of antioxidants from diet and average years of antioxidant supplementation. The average intakes of beta-carotene, vitamin C, vitamin E, selenium and zinc from diet and average years of supplementation were similar for cases and controls for both premenopausal and postmenopausal women.

**Table 2:** Average intakes of antioxidants by case-control status, NECSS, Canada, 1994-1997

	Premenopausal			Postmenopausal		
		
Characteristic	Cases (N = 866)	Controls (N = 845)	*p*	Cases(1496)	Controls (n = 1617)	*P *
	Mean	Mean		Mean	Mean	
From diet						
Beta-carotene (μg/wk)	31643.6	32369.8	0.55	37831	37648.6	0.89
Vitamin E (mg/wk)	56.4	56.8	0.82	64.2	62.6	0.37
Vitamin C (mg/wk)	1101.2	1137.1	0.38	1184.8	1151.3	0.26
Selenium (μg/wk)	660.2	651.6	0.54	662.9	656.9	0.65
Zinc (mg/wk)	65.7	64.9	0.52	67.1	65.7	0.27
From supplement (years)						
Vitamin A	0.88	1.02	0.38	1.02	1.06	0.76
Beta-carotene	0.59	0.77	0.16	0.68	0.76	0.39
Vitamin C	3.22	3.53	0.24	3.85	3.68	0.44
Vitamin E	1.53	1.80	0.16	2.61	2.72	0.56
Zinc	0.60	0.83	0.09	0.57	0.71	0.14
Selenium	0.37	0.47	0.33	0.41	0.41	0.95
						

Table 3 presents the breast cancer risk associated with different levels of antioxidants from dietary intake including beta-carotene, alpha-carotene, lycopene, lutein and zeaxanthin, vitamin C, vitamin E, selenium and zinc. No significant association was observed between breast cancer risk and the highest quartiles of these antioxidant intakes from dietary sources compared with the lowest quartiles of intake, either for premenopausal or for postmenopausal breast cancer.

**Table 3:** ORs of breast cancer associated with intake of dietary antioxidants, NECSS, Canada, 1994-1997

	Pre-menopausal					Post-menopausal				
		
Nutrient	Quartiles of intake					Quartiles of intake				
	Q1*	Q2	Q3	Q4	*p *for	Q1*	Q2	Q3	Q4	*p *for
		OR† (95% CI)	OR† (95% CI)	OR† (95% CI)	trend		OR†† (95% CI)	OR†† (95% CI)	OR†† (95% CI)	trend
Carotenoids										
Beta-carotene (μg/wk)#	1.00	0.87 (0.65-1.15)	0.97 (0.73-1.28)	0.87 (0.65-1.17)	0.55	1.00	0.89 (0.72-1.10)	0.84 (0.68-1.04)	0.85 (0.68-1.06)	0.13
Alpha-carotene (μg/wk)	1.00	0.82 (0.61-1.10)	0.95 (0.72-1.25)	0.85 (0.64-1.14)	0.46	1.00	0.86 (0.72-1.11)	0.89 (0.71-1.12)	0.92 (0.73-1.14)	0.53
Lycopene (μg/wk)	1.00	0.97 (0.74-1.28)	0.99 (0.76-1.31)	0.88 (0.65-1.20)	0.53	1.00	1.15 (0.93-1.43)	1.20 (0.97-1.49)	1.21 (0.96-1.51)	0.14
Lutein and Zeaxanthin (μg/wk)	1.00	0.92 (0.69-1.23)	1.08 (0.82-1.44)	0.82 (0.61-1.11)	0.41	1.00	0.81 (0.64-1.02)	0.94 (0.75-1.19)	0.94 (0.74-1.19)	0.86
Beta cryptozanthin (μg/wk)	1.00	1.20 (0.89-1.60)	1.15 (0.87-1.53)	1.18 (0.86-1.60)	0.38	1.00	1.21 (0.98-1.50)	1.13 (0.90-1.40)	1.31 (1.04-1.65)	0.05
Vitamin C (mg/wk) #	1.00	1.00 (0.75-1.35)	1.21 (0.92-1.61)	0.87 (0.64-1.20)	0.89	1.00	1.13 (0.91-1.40)	1.14 (0.91-1.42)	1.24 (0.98-1.56)	0.09
Vitamin E (mg/wk) #	1.00	1.26 (0.93-1.70)	1.22 (0.89-1.68)	0.88 (0.62-1.25)	0.43	1.00	0.93 (0.75-1.15)	0.93 (0.74-1.17)	0.92 (0.73-1.17)	0.54
Selenium (μg/wk) #	1.00	1.22 (0.91-1.63)	1.23 (0.90-1.68)	1.10 (0.75-1.61)	0.58	1.00	1.21 (0.99-1.53)	1.10 (0.88-1.39)	1.09 (0.84-1.43)	0.73
Zinc (mg/wk) #	1.00	1.05 (0.78-1.41)	1.14 (0.83-1.57)	1.18 (0.79-1.77)	0.35	1.00	1.07 (0.85-1.35)	1.07 (0.84-1.36)	1.21 (0.91-1.71)	0.23

* Reference category

† Adjusted for age, province of residence, education, smoking pack years, alcohol consumption, number of live births, age at menarche, and total energy intake.

†† Adjusted for age, province of residence, education, smoking pack years, alcohol consumption, BMI, recreational physical activity, number of live births, age at menarche, and total energy intake.

# Also adjusted for years of taking antioxidants from supplementation.

Table 4 shows the breast cancer risk associated with different categories (years) of antioxidants from supplementation. For premenopausal breast cancer, supplementation of 10 years or longer was associated with statistically significant reduction in breast cancer risk for zinc. For postmenopausal breast cancer, supplementation of 10 years or longer was associated with statistically significant reduction in breast cancer risk for multiple vitamin, beta-carotene, vitamin C, vitamin E and zinc. No significant association between antioxidants and risk of breast cancer, in either premenopausal or postmenopausal cases, was observed, for supplementation less than 10 years prior to data collection, however.

**Table 4:** ORs of breast cancer associated with supplementation of antioxidants, NECSS,Canada,1994-1997

	Premenopausal				Postmenopausal			
		
Variable	Cases	Controls	OR † (95% CI)	*p *trend	Cases	Controls	OR†† (95% CI)	*p *trend
Multiple vitamin (years)				0.53				0.04
0 (never taken)*	269	249	1.00		654	731	1.00	
≤ 2 year	282	294	0.86 (0.67-1.10)		312	335	0.91 (0.75-1.11)	
3-9 year	180	177	0.85 (0.64-1.13)		292	268	1.03 (0.83-1.27)	
≥ 10 years	135	125	0.93 (0.69-1.27)		238	283	0.74 (0.59-0.92)	
Vitamin A (years)				0.26				0.31
0 (never taken)*	690	677	1.00		1235	1350	1.00	
≤ 2 year	105	95	1.02 (0.75-1.39)		127	133	0.90 (0.69-1.19)	
3-9 year	47	40	1.03 (0.65-1.61)		72	61	1.12 (0.77-1.63)	
≥ 10 years	24	33	0.61 (0.35-1.06)		62	73	0.77 (0.53-1.11)	
Beta-carotene (years) #				0.86				0.15
0 (never taken)*	675	689	1.00		1219	1348	1.00	
≤ 2 year	144	93	1.54 (1.16-2.06)		182	162	1.01 (0.79-1.29)	
3-9 year	35	42	0.85 (0.53-1.37)		63	60	1.01 (0.69-1.49)	
≥ 10 years	12	21	0.51 (0.24-1.06)		32	47	0.58 (0.36-0.95)	
Vitamin C (years) #				0.26				0.07
0 (never taken)*	337	357	1.00		687	792	1.00	
≤ 2 year	267	223	1.20 (0.94-1.53)		306	317	0.86 (0.70-1.05)	
3-9 year	165	148	1.08 (0.82-1.43)		268	261	0.96 (0.77-1.19)	
≥ 10 years	97	117	0.75 (0.54-1.03)		235	247	0.79 (0.63-0.99)	
Vitamin E (years) #				0.25				0.05
0 (never taken)*	493	513	1.00		822	935	1.00	
≤ 2 year	245	171	1.46 (1.15-1.85)		316	286	1.05 (0.86-1.28)	
3-9 year	89	113	0.73 (0.53-1.01)		209	220	0.91 (0.72-1.15)	
≥ 10 years	39	48	0.74 (0.47-1.17)		149	176	0.75 (0.58-0.97)	
Zinc (years) #				0.004				0.02
0 (never taken)*	736	688	1.00		1297	1420	1.00	
≤ 2 year	85	95	0.81 (0.58-1.11)		120	94	1.14 (0.84-1.53)	
3-9 year	27	33	0.73 (0.43-1.25)		52	56	0.83 (0.55-1.26)	
≥ 10 years	18	29	0.46 (0.25-0.85)		27	47	0.47 (0.28-0.78)	
Selenium (years) #				0.59				0.85
0 (never taken)*	755	754	1.00		1355	1504	1.00	
≤ 2 year	85	50	1.65 (1.13-2.40)		88	56	1.43 (0.98-2.05)	
3-9 year	16	27	0.59 (0.31-1.12)		29	30	0.90 (0.52-1.57)	
≥ 10 years	10	14	0.57 (0.25-1.34)		24	27	0.75 (0.42-1.34)	

* Reference category

† Adjusted for age, province of residence, education, smoking pack years, alcohol consumption, number of live births, age at menarche, and total energy intake.

†† Adjusted for age, province of residence, education, smoking pack years, alcohol consumption, BMI, recreational physical activity, number of live births, age at menarche, and total energy intake.

# also adjusted for antioxidant intake from diet.

Table 5 gives the odds ratios of breast cancer associated with high intakes of beta-carotene, vitamin C, vitamin E, zinc, and selenium from diet only, from supplementation only and from both sources. High intakes of beta-carotene, vitamin C, vitamin E, Zinc, and selenium from diet only were not significantly associated with breast cancer risk compared with low intake of these antioxidants for both premenopausal and postmenopausal women. Results for high intake of these antioxidants from supplementation only were similar to table 3. High intakes from both sources were not significantly associated with breast cancer risk except for vitamin C in premenopausal women and for zinc in postmenopausal women.

**Table 5:** ORs of breast cancer associated with total intake of antioxidants, NECSS,Canada,1994-1997

	Premenopausal				Postmenopausal			
		
Variable	Cases	Controls	OR † (95% CI)	*p *	Cases	Controls	OR†† (95% CI)	*p *
Beta-carotene								
From Diet only								
Low or no intake	423	435	1.00		781	837	1.00	
High intake *	443	410	1.09 (0.88-1.34)	0.43	715	780	0.96 (0.82-1.13)	0.66
From supplementation only								
Low or no intake	864	836	1.00		1487	1598	1.00	
High intake**	2	9	0.11 (0.01-0.88)	0.04	9	19	0.47 (0.20-1.08)	0.07
From both sources								
Low or no intake	856	833	1.00		1473	1589	1.00	
High intake***	10	12	0.77 (0.32-1.87)	0.57	23	28	0.69 (0.37-1.27)	0.23
Vitamin C (years)								
From Diet only								
Low or no intake	467	489	1.00		861	962	1.00	
High intake *	399	356	1.23 (0.99-1.53)	0.06	635	655	1.24 (0.99-1.42)	0.07
From supplementation only								
Low or no intake	823	794	1.00		1405	1524	1.00	
High intake**	43	51	0.70 (0.44-1.10)	0.12	91	93	0.85 (0.61-1.17)	0.32
From both sources								
Low or no intake	812	779	1.00		1352	1463	1.00	
High intake***	54	66	0.65 (0.44-0.98)	0.04	144	154	0.78 (0.60-1.03)	0.08
Vitamin E (years)								
From Diet only								
Low or no intake	451	454	1.00		837	922	1.00	
High intake *	415	391	0.98 (0.78-1.22)	0.85	659	695	1.06 (0.89-1.25)	0.53
From supplementation only								
Low or no intake	852	828	1.00		1451	1555	1.00	
High intake**	14	17	0.72 (0.33-1.59)	0.42	45	62	0.64 (0.42-0.99)	0.04
From both sources								
Low or no intake	841	814	1.00		1392	1503	1.00	
High intake***	25	31	0.71 (0.40-1.25)	0.23	104	114	0.86 (0.64-1.17)	0.35
Zinc (years)								
From Diet only								
Low or no intake	430	451	1.00		747	855	1.00	
High intake *	436	394	1.13 (0.89-1.45)	0.32	749	762	1.13 (0.94-1.35)	0.20
From supplementation only								
Low or no intake	859	830	1.00		1487	1597	1.00	
High intake**	7	15	0.29 (0.10-0.83)	0.02	9	20	0.41 (0.18-0.96)	0.04
From both sources								
Low or no intake	855	831	1.00		1478	1590	1.00	
High intake***	11	14	0.73 (0.31-1.72)	0.47	18	27	0.51 (0.27-0.97)	0.04
Selenium (years)								
From Diet only								
Low or no intake	417	426	1.00		747	822	1.00	
High intake *	449	419	1.01 (0.79-1.29)	0.93	749	795	0.99 (0.82-1.18)	0.88
From supplementation only								
Low or no intake	861	834	1.00		1488	1604	1.00	
High intake**	5	11	0.39 (0.13-1.16)	0.09	8	13	0.50 (0.19-1.32)	0.16
From both sources								
Low or no intake	861	842	1.00		1480	1603	1.00	
High intake***	5	3	1.77 (0.33-9.69)	0.51	16	14	0.82 (0.37-1.80)	0.61

† Adjusted for age, province of residence, education, smoking pack years, alcohol consumption, number of live births, age at menarche, and total energy intake.

†† Adjusted for age, province of residence, education, smoking pack years, alcohol consumption, BMI, recreational physical activity, number of live births, age at menarche, and total energy intake.

* In comparison with low intake (lower than median intake).

** In comparison with low intake (less than 10 years of supplementation).

*** In comparison with not high intake from both sources.

## Discussion

Our large population based study indicated that supplementations of 10 years or longer with zinc were associated with statistically significant reductions in premenopausal breast cancer risk. For postmenopausal women, supplementations of 10 years or longer of multiple vitamins, beta-carotene, vitamin C, vitamin E and zinc were associated with statistically significant reductions for breast cancer risk. No significant difference was observed in breast cancer risk, either premenopausal or postmenopausal, across different levels of antioxidants from dietary sources, nor was there a significant effect of supplementation shorter than 10 years. With the ability to draw a national sample, our study is amongst the largest in the field. We have also been able to adjust for a number of risk factors known to be related to breast cancer such as BMI, energy intake, and physical activity. However, this study could not determine the overall effect of total dose or intake of antioxidants from both diet and supplement.

Carotenoids (precursor of vitamin A) include alpha-carotene, beta-carotene, lutein, lycopene, and beta-cryptoxanthin. Higher dietary intakes and blood concentrations of carotenoids, including beta-carotene, have been shown to reduce breast cancer risk, but the results are not entirely consistent [8,19]. A summarized analysis of 8 case-control studies showed a 15% reduction in breast cancer risk associated with the highest category of dietary beta-carotene [20]. A meta-analysis of 11 studies published from 1982 to 1997 also suggests that intake of 7000 μg per day or more of beta-carotene was associated with 18% reduction of breast cancer risk compared with intake of 1000 μg per day or less [21]. In addition, a nested case-control study reported a 50% decrease in breast cancer risk in women with high levels of serum beta-carotene, lycopene, and total carotene compared with those with low levels of these micronutrients [22]. Higher serum beta-carotene concentration was also found to be associated with lower breast cancer risk in other cohort studies [23-26] and case-control studies [27,28]. However, no association was observed in some studies for serum beta-carotene [29,30] and for dietary beta-carotene, alpha-carotene, lutein, beta-cryptoxanthin and lycopene [12,31] and in one randomized trail on beta-carotene supplementation use [13]. The Women's Health Initiative Observational Study on postmenopausal women also suggests that the association between breast cancer risk and carotenoids differs by estrogen receptor (ER) and progesterone receptor (PR) status since this study found that dietary alpha-carotene and beta-carotene were inversely associated with the risk of ER+PR+ breast cancer only, but not with other breast cancer groups defined by ER and PR status [32]. Carotenoids have been shown to protect against DNA alterations via a decrease in oxidative and other damage to DNA in humans [3,6,7,11] and protect cells in vitro against neoplastic transformation [3,11].

Zinc is involved with metallothionine synthesis, which is believed to inhibit free radical production [33]. Zinc supplementation has also been shown to reduce oxidative stress and DNA strand breaks and early and delayed apoptosis, as well as improve immune function [33]. Two case- control studies [34,35] demonstrated statistically significant inverse associations between Zn exposure, measured in serum and diet, respectively, and breast cancer risk. However, two nested case- control studies [36,37] reported no association between toenail Zn or zinc in benign breast tissue and breast cancer risk. Another case-control study also found no difference in either hair or plasma level of zinc between cases and controls [38]. The use of different status biomarkers in these studies may explain partly the mixed results of different studies [39].

Although studies in cell cultures and animals showed that vitamin E and C prevent transformation of normal cells to cancer cells and selectively induce apoptosis in cancer cells, the results of epidemiologic studies have been inconsistent [19]. The review of 12 cohort studies on dietary vitamin C and vitamin E intake and the incidence of breast cancer suggested no consistent association between both vitamin and breast cancer incidence among these studies [2]. The Danish Diet Cancer and Health Cohort observed a significant inverse association for total vitamin E (including dietary and supplemental) intake among postmenopausal women (RR = 0.59, 95% CI: 0.37-0.95) [40]. An inverse association between breast cancer risk and high serum level of alpha-tocopherol (vitamin E) was also found in 3 cohort studies [24,26,41]. However, several cohort studies reported no significant association [22,29,30,32,42-45]. The evidence for vitamin C is also inconsistent. A pooled analysis of 9 case-control studies found a 31% decrease in breast cancer risk associated with each 300 mg of vitamin C per day [20]. A meta-analysis of 9 case-control and cohort studies observed a summary RR of 0.80 (95% CI: 0.68-0.95) for high consumption compared with low consumption [21]. On the other hand, several cohort studies reported no significant association between vitamin C and breast cancer risk [32,42-45]. One randomized controlled trial found no relationship of vitamin C and E supplementation use with breast cancer risk [13].

Because of differences in study population, study design, and nutritional assessment, it is rather difficult to reconcile results from different studies. Our study did not find any association between any antioxidant from dietary sources and breast cancer risk, or an effect of supplementation shorter than 10 years on breast cancer risk, for both premenopausal and postmenopausal cases. It is difficult to measure nutrients from dietary intake by case control studies. Although the response rate was 69.7% for breast cancer cases, selection (inclusion) of cases was not related to exposure status (i.e. antioxidant intake), therefore, selection bias is unlikely to occur. Recall bias, inherent in all case-control design, is possible because cases might bias their responses to questions on diet after a few months of cancer diagnosis. The possibility that such a bias was introduced into our study was reduced by including many questions on other exposures, such as socio-demographic factors, physical activity, weight, smoking history, employment history and residential history and by not placing any particular emphasis on diet in the questionnaire. Although we used a widely validated questionnaire, measurement error of dietary intakes, for example, self-reports of long-term dietary supplements, could introduce misclassification in exposure status, but it is likely to be nondifferential. The 69-item food frequency questionnaire might fail to capture all kinds of food consumed and underestimated total dietary intake. The time window of exposure is also critical in epidemiologic studies of diseases with long incubation period such as breast cancer. To improve accuracy and precision, we used two year from diagnosis as the point of measurement in the dietary survey. Another limitation is that we could not assess the dose of antioxidants from supplementation because doses of antioxidants from different producers, years and batches may be different. This might also affect accuracy and precision of our results. Antioxidants, either from dietary intake or from supplementation, may exert their effect over a long period of time. That may be the reason why we observed the effect for supplementation of 10 years or longer only. In addition, we could not adjust for the family history of breast cancer, benign breast disease, oral contraceptive use, hormone replacement therapy, and hormone receptor status in the analyses because the information was not available for all records. Furthermore, some of the risk estimates were based on small numbers of subjects, especially for some categories of supplementation; therefore, some of the significant associations may be due to due to chance.

The recommended intakes (recommended dietary allowance) for adult women are 700 μg/day for vitamin A, 15 mg/day for vitamin E, 75 mg/day for vitamin C, 55 μg/day for selenium and 8 mg/day for zinc [46]. Canadian nutrition survey in 2004 data showed that percentages of adult women 19 years or over with a usual intake below the estimated average requirement were 35.8% for vitamin A, 16.7% for vitamin C and 14.0% for zinc [47]. Our data showed that the average dietary intake for the study population had reached the recommended dietary allowance for vitamin C, selenium and zinc, but was lower for vitamin E.

## Conclusion

In summary, our population-based study showed that supplementation of 10 years or longer of certain antioxidants such as beta-carotene, vitamin C, vitamin E and zinc may reduce the risk of breast cancer, although this study could not determine the overall effect of total dose or intake of antioxidants from both diet and supplement. Further investigations are warranted to confirm our results. The findings of our study may shed light on the prevention and control of breast cancer.

## Competing interests

The authors declare that they have no competing interests.

## Authors' contributions

The Canadian Cancer Registries Epidemiology Research Group contributed to data collection. SYP conceived the study, performed the analyses, wrote the manuscript and incorporated input from all other authors on the manuscript. JZ carried out part of the analyses and reviewed the literature. SWW provided guidance on data analysis and provided critical comments on the manuscript. LG, and HM directed the overall study and provided critical comments on the manuscript. All authors have read and approved the final version of the manuscript.

## Pre-publication history

The pre-publication history for this paper can be accessed here:

http://www.biomedcentral.com/1471-2407/11/372/prepub
